# Treatise on Human Physiology for the Use of Students and Practitioners of Medicine

**Published:** 1887-12

**Authors:** 


**Treatise on Human Physiology for the Use of Students and Practitioners of Medicine.** By HENRY C. CHAPMAN, M. D., Professor of Institutes of Medicine and Medical Jurisprudence, in the Jefferson Medical College, etc. Philadelphia: Lea Brothers & Co. 1887.

Prof. Chapman's abilities as a teacher have been long recognized, and this book is the outcome of years of study and observation, and long experience in this special branch of medicine. It represents very fully the existing state of physiology, based upon comparative and pathological anatomy, clinical medicine, physics and chemistry, as well as upon experimental research. While we have already many excellent works on the subject, the present one has a special value to the student and practitioner, as devoted less to the discussion of theories and more to the practical application of the well-ascertained truths which the advance of science have given to the profession in this department, which may be considered the foundation of rational medicine. The work is issued in very handsome form by the Lea's.

